# Pan-cancer analysis reveals the associations between MMP13 high expression and carcinogenesis and its value as a serum diagnostic marker

**DOI:** 10.18632/aging.204599

**Published:** 2023-03-22

**Authors:** Xinhui Zhang, Qingmei Deng, Xiaofeng Wan, Jingyu Zhao, Xin Zheng, Hongzhi Wang, Hong-Qiang Wang, Wulin Yang

**Affiliations:** 1 School of Basic Medical Sciences, Anhui Medical University, Hefei 230032, China; 2 Anhui Province Key Laboratory of Medical Physics and Technology, Institute of Health and Medical Technology, Hefei Institutes of Physical Science, Chinese Academy of Sciences, Hefei 230031, China; 3 Medical Pathology Center, Hefei Cancer Hospital, Chinese Academy of Sciences, Hefei 230031, China; 4 Institutes of Physical Science and Information Technology, Anhui University, Hefei 230601, China; 5 State Key Laboratory of Oncology in South China, Collaborative Innovation Center for Cancer Medicine, Sun Yat-sen University Cancer Center, Guangzhou 510060, China; 6 Biological Molecular Information System Laboratory, Institute of Intelligent Machines, Hefei Institutes of Physical Science, Chinese Academy of Sciences, Hefei 230031, China

**Keywords:** MMP13, pan-cancer, prognosis, diagnosis, biomarker

## Abstract

Background: Matrix metalloproteinase-13 (MMP13) is a member of the endopeptidase matrix metalloproteinase family, which is involved in many normal physiological processes and even tumorigenesis. However, its co-carcinogenic signature in different cancers is not fully understood.

Methods: In this study, we first analyzed the expression of MMP13 in pan-cancer and its association with prognosis, immune infiltration, and cancer-related signaling pathways through integrated bioinformatics. Furthermore, western blotting (WB) was used to verify the expression of MMP13 and epithelial-mesenchymal transition (EMT) factors in cancer tissues. Finally, the value of MMP13 as a serum diagnostic marker was analyzed by enzyme-linked immunosorbent assay (ELISA).

Results: MMP13 expression is frequently upregulated in multiple cancers that always indicate an adverse prognosis. MMP13 undergoes comprehensive genetic alterations and promoter methylation reduction in various tumors. Additionally, immune correlation analysis showed that MMP13 expression was significantly associated with TMB, MSI, and tumor immune infiltration. Pathway enrichment analysis showed that MMP13 upregulation was correlated with activation of the EMT signaling pathway, which was verified by WB in lung adenocarcinoma tissues. Most importantly, ELISA results showed that serum MMP13 levels could be used for the diagnosis of multiple tumors, including BRCA, HNSC, LUAD, and LUSC, with the area under the curve (AUC) values of 0.8494, 0.9259, 0.7144, and 0.8575, respectively.

Conclusions: MMP13 is often overexpressed across cancers and predicts poor prognosis, with the potential as a therapeutic target. Furthermore, the up-regulation of its expression can be effectively reflected in the serum protein level, thus serving as a valuable diagnostic marker.

## INTRODUCTION

Cancer is ranked as a leading cause of death and an important barrier to increasing life expectancy in every country in the world [1]. Despite continued improvements in the methods of diagnosis and treatment of cancers, the burden of cancer incidence and mortality is still growing rapidly worldwide [2]. Therefore, it is urgent to find novel targets for cancer treatment and novel-sensitive tumor biomarkers for the diagnosis of cancer. The diagnosis of cancer often requires invasive tests, such as pathological biopsies, that are not readily accepted by patients. And these tests are often only effective for late-stage disease, leading to patients missing the best opportunities for treatment. Data mining through the analysis of The Cancer Genome Atlas (TCGA) and other public databases, combined with the validation of clinical samples, is an effective way to comprehensively understand the functions of some genes in cancer and their significance for cancer diagnosis and treatment [3].

Matrix metalloproteinases (MMPs) are members of zinc-dependent endopeptidases and are key regulators of tissue reorganization that occurs in wound healing, inflammatory responses, and malignancy [4]. Matrix metalloproteinase-13 (MMP13), also termed collagenase-3, is a member of the matrix metalloproteinase family of endopeptidases and displays extremely high gelatinolytic activity [5]. A large number of studies have shown that MMP-13 is often overexpressed in tumors, such as nasopharyngeal cancer [6], cutaneous squamous cell carcinoma [7], gastric cancer [8], breast cancer [9], and head and neck squamous cell carcinoma [10], making it a potential diagnostic and therapeutic target [11]. MMP13 is also a crucial factor in invasiveness, metastasis, and prognosis in tumor tissues [12–15]. It has also been reported that MMP13 can degrade several ECM components and plays a role as a stromal mediator in controlling persistent angiogenesis in skin carcinoma [16, 17]. Additionally, MMP13 is involved in the degradation of articular cartilage not only in osteoarthritis but also in rheumatoid arthritis [18]. Due to numerous MMP members and functional redundancy among them, comprehensive studies on MMP13 expression, genetic alterations, and biological function are required from the perspective of pan-cancer to judge their value as tumor markers.

In this study, we performed a pan-cancer analysis of MMP13 based on 33 human cancers. Through a variety of databases and web-based tools, we comprehensively analyzed the changes in MMP13 expression in tumors and assessed the correlation between MMP13 expression and prognosis, tumor mutation burden (TMB), microsatellite instability (MSI), DNA methylation, tumor microenvironment, and immune-related genes in multiple cancer types. We also analyzed the relationship between the expression of MMP13 and the activity of cancer-related pathways in which MMP13 might be involved. Finally, we used ELISA assay to determine the diagnostic value of MMP13 in the serum of specific tumors such as non-small cell lung cancer. We hope that this study will provide new insights into the role of MMP13 in the genesis, diagnosis, and prognosis of human cancers.

## MATERIALS AND METHODS

### Analysis of MMP13 expression across human cancers

RNA-seq data for 33 cancer types were obtained from the TCGA database (https://portal.gdc.cancer.gov/) [19]. The differential expression of MMP13 in tumor and normal tissues was compared by Wilcoxon’s test. The R package “ggpubr” was used to draw the graph. We further analyzed the expression differences using the GEPIA2 (http://gepia2.cancer-pku.cn/) online tool [20], including the RNA-seq expression data of 9,736 tumors and 8,587 normal samples from the TCGA and the GTEx projects. The Cancer Cell Line Encyclopedia (https://sites.broadinstitute.org/ccle) [21] database was used to collect MMP13 expression data in 33 types of primary tumor cell lines.

### Survival analysis of MMP13

Gene Set Cancer Analysis (GSCA, http://bioinfo.life.hust.edu.cn/GSCA/#/) [22] is an integrated platform for genomic, pharmacogenomic, and immunogenomic gene set cancer analysis. We used GSCA to analyze the relationship between MMP13 expression and survival. We selected four indicators, overall survival (OS), disease-specific survival (DSS), disease-free interval (DFI), and progression-free interval (PFI), to study the relationship between MMP13 expression and patient prognosis. The Kaplan–Meier curves were plotted for accordingly tumors with significant prognosis using the R packages “survival” and “survminer.”

### DNA methylation analysis of MMP13

We used GSCA to analyze MMP13 methylation differences between tumors and adjacent normal tissues and investigated the correlation between MMP13 methylation and prognosis, including OS, DSS, DFI, and PFI. Simultaneously, correlation analysis between MMP13 mRNA expression and gene promoter methylation was performed for each tumor, which was also done on the GSCA website.

Finally, the DNA methylation level of MMP13 in LUSC and the relationships between the expression of MMP13 and clinical features of LUSC were explored via the MEXPRESS website (https://mexpress.be/) [23, 24]. MEXPRESS is a data visualization tool designed for the easy visualization of TCGA expression, DNA methylation, and clinical data, as well as the relationships between them.

### Genomic mutation of MMP13 in cancers

The cBio cancer genomics portal (cBioPortal, https://www.cbioportal.org/) [25] is an open platform for exploring multidimensional cancer genomics data. Based on the cBioPortal, we acquired the protein structure schematic of the overall mutation site information of MMP13 and selected the mutation type and alteration frequency for alteration analysis of MMP13 status in different tumor types. By GSCA website, we assessed the correlation between mRNA expression quantity of MMP13 and CNV in different tumors and the relationship between the single nucleotide variation (SNV) and the copy number variation (CNV) of MMP13 and tumor prognosis.

### Association of MMP13 expression with tumor mutation burden and tumor microsatellite instability

Tumor mutational burden (TMB) is a measure of the number of mutations in one cancer [26] and is a potential biomarker that can predict the response to immunotherapy [27]. Microsatellite instability (MSI) is a molecular tumor phenotype resulting from genomic hypermutability, expressed as nucleotides of repeating DNA fragments being added or lost, which may also influence immune checkpoint therapy [28]. TMB and MSI scores were calculated, as well as their association with MMP13 expression. The results are generated as a radar chart using the R package “fmsb.”

### Immune factors correlation analysis

The immune score, stromal score, and estimate score of 33 types of TCGA cancer samples were calculated from the “estimate” R package and “limma” R package. Then, transcript gene expression data were then intersected with these scores for Spearman correlation tests. The immune subtype data were downloaded from UCSC xena (https://xenabrowser.net/datapages/). We used the “limma,” “ggplot2,” and “reshape2” R packages for the immune subtype analysis. Furthermore, we evaluated the association between MMP13 expression and the immune-related genes. The result is presented as a heatmap that was visualized using the “reshape2” and “RColorBrewer” packages.

### Pathway activity analysis and protein-protein interaction (PPI) network construction

The association between MMP13 expression and activity of cancer-related pathways in human cancers was analyzed on the GSCA website. GeneMANIA (http://www.genemania.org) [29, 30] is an interactive website that uses huge functional association data, including protein and genetic interactions, pathways, co-expression, co-localization, and protein structural domain similarities to discover genes with similar functions. GeneMANIA was applied to PPI analysis in this study.

### Specimen collection

We collected 7 paired tissue samples from lung adenocarcinoma patients. Serum samples were collected from 27 healthy subjects, 30 BRCA cases, 30 HNSC cases, 38 LUAD cases, and 26 LUSC cases. Specimens were obtained from the Hefei Cancer Hospital, Chinese Academy of Sciences. The study procedures were approved by the Institutional Review Board of the Hefei Institutes of Physical Science, Chinese Academy of Sciences (CAS) (SWYX-Y-2022-39) and the Hefei Cancer Hospital, CAS (SL-KY2021-016). Blood samples were collected before any form of medical intervention, then centrifuged at 2,000 × g for 20 min to obtain serum. All samples were stored at −80°C before the experiments.

### Western blot

Tissue proteins were extracted using the RIPA Buffer (SparkJade) and protease inhibitor (SparkJade). Proteins were denatured by boiling in SDS-PAGE protein loading buffer (SparkJade). Total proteins were subjected to 5–10% sodium dodecyl sulfate-polyacrylamide gel electrophoresis (SDS-PAGE). Proteins were transferred to immobilon-P PVDF membranes (MILLIPORE) using an immunoblot transfer buffer. After incubation at 5% BSA (Biofroxx) for 1 h at room temperature, membranes were incubated with primary antibodies overnight at 4°C. Antibodies purchased from ZEN-BIOSCIENCE (E-cadherin, N-cadherin), Cell Signaling (Vimentin), Proteintech (MMP13), Transgen (GAPDH) were used according to manufacturer’s recommendations. The next day, membranes were incubated with horseradish peroxidase-conjugated anti-mouse or anti-rabbit secondary antibodies (SparkJade or ZEN-BIOSCIENCE) for 1 h at room temperature. After three washing steps, blots were stained using a chemiluminescence system (ECL, YEASEN) and exposed to X-ray film. Details of antibodies are shown in Supplementary Table 1.

### ELISA

Serum biomarker levels were determined using a double-antibody sandwich ELISA according to the manufacturer’s instructions (MMP13, CUSABIO). Briefly, 100 μl of the test samples were added and incubated for 2 h at 37°C. Subsequently, 100 μl/well of the biotin-antibody was added and incubated for 1 h at 37°C. Next, 100 μl/well of HRP-avidin was added and incubated for 1 h at 37°C. Finally, the substrate solution (tetramethyl benzidine) was added, and the reaction was stopped with H_2_SO_4_ and read at an OD of 450 nm.

### Statistical analysis

Gene expression in normal tissue and tumor tissue was compared across cancers using the Wilcoxon test. Survival analyses were performed using the log-rank test by the Kaplan–Meier method or the cox regression model. The Spearman or Pearson method was used to study the correlation between two variables. The comparisons of each protein concentration between the different groups were assessed using the Mann-Whitney test. The diagnostic efficiency of each protein was evaluated by the area under the curve (AUC). *P*-value < 0.05 was considered statistically significant. Statistical analyses were performed using GraphPad Prism 9 or R software.

### Availability of data and materials

The datasets analyzed in the current study are available in the TCGA repository. (https://gdc.cancer.gov/). Relevant data or materials can also be obtained from the corresponding author upon reasonable request.

## RESULTS

### MMP13 mRNA levels were upregulated in multiple cancers

**Figure 1:**
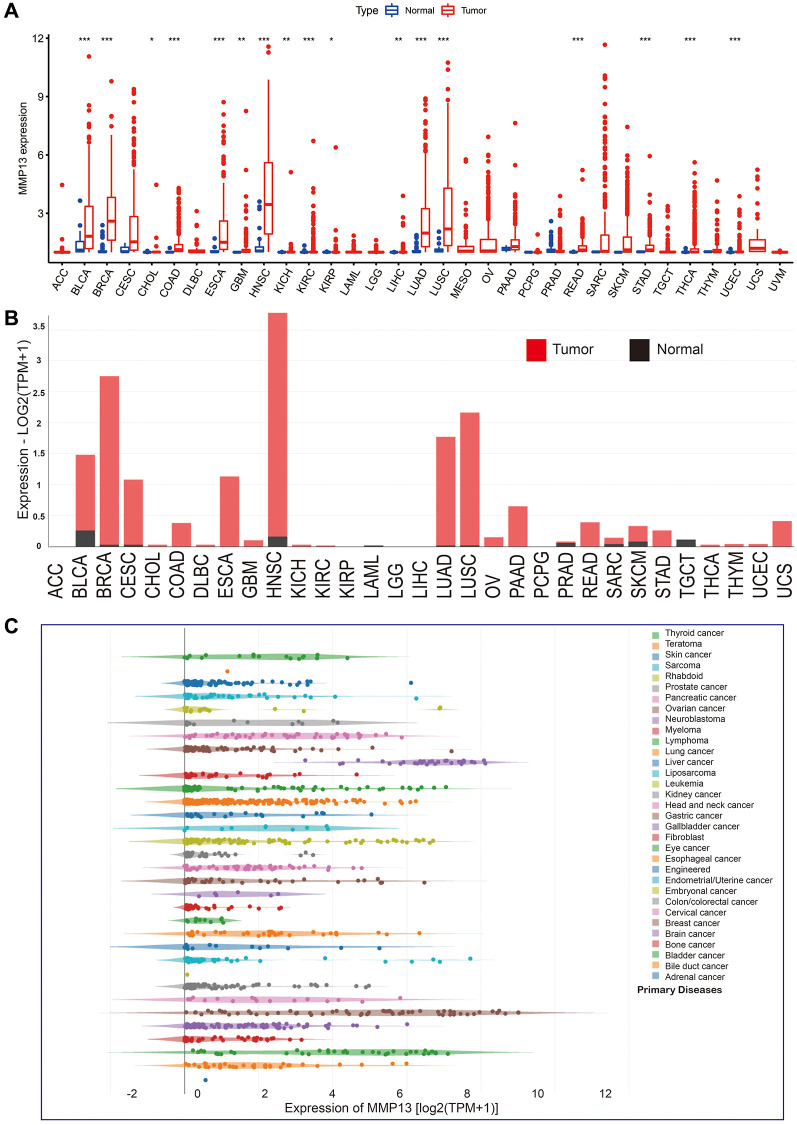
**mRNA expression levels of MMP13 in pan-cancer.** (**A**) The levels of MMP13 expression in different types of cancers were analyzed based on the TCGA database. The blue boxplots indicate the normal tissues. The red boxplots indicate the cancer tissues. ^*^P < 0.05, ^**^P < 0.01, ^***^P < 0.001. (**B**) Analysis of MMP13 mRNA expression in different cancers by the GEPIA2 web tool. (**C**) MMP13 mRNA expression in 33 tumor cell lines from the CCLE database.

We analyzed the expression level of MMP13 mRNA across all 33 cancer types available in the TCGA database (Figure 1A). The results showed that the expression of MMP13 was generally higher in tumor tissues than in normal tissues, with a significant difference in BLCA, BRCA, COAD, ESCA, HNSC, KIRC, LUAD, LUSC, READ, STAD, THCA, UCEC, GBM, KICH, LIHC, CHOL, KIRP. To further confirm the upregulation of MMP13 mRNA in different cancers, we analyzed relevant data from the GEPIA database. Compared to normal tissues, MMP13 expression levels were increased in BLCA, BRCA, CESC, COAD, ESCA, HNSC, LUAD, LUSC, PAAD, READ, SKCM, STAD, and UCS (Figure 1B). Furthermore, we compared the expression of MMP13 in 33 tumor cell lines using the CCLE database, and the results showed that MMP13 was highly expressed in many cancer cell lines. (Figure 1C).

The above results indicate that MMP13 is upregulated in multiple tumor tissues and may be an oncogene in human cancers.

### Relationship between the expression of MMP13 and prognosis in human cancers

**Figure 2:**
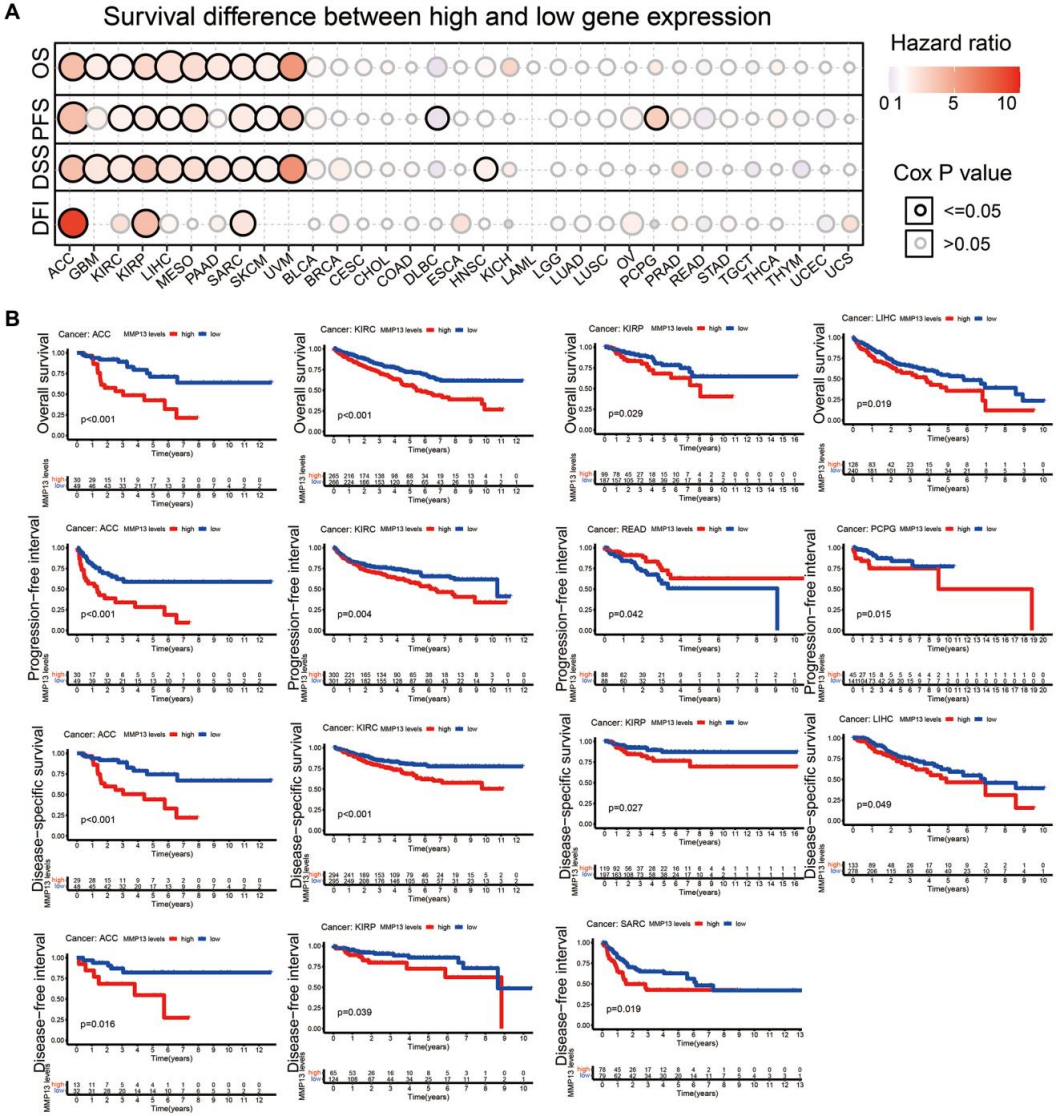
**Correlation between MMP13 expression and prognosis.** (**A**) Analysis of the relationship between MMP13 mRNA expression and patient survival in various cancers, including OS, PFS, DSS, and DFI. (**B**) Kaplan-Meier survival curve showed that MMP13 gene expression was significantly associated with survival in patients with certain types of cancer, including ACC, KIRC, KIRP, and LIHC. Abbreviations: OS: overall survival; PFS: progression-free survival; DSS: disease-specific survival; DFI: disease-free interval.

To investigate the prognostic value of MMP13 upregulation in different tumors, we used the GSCA database to perform OS, PFS, DSS, and DFI analyses on the relationship between MMP13 expression and tumor prognosis (Figure 2A). High expression of MMP13 was associated with poor OS prognosis in ACC (*P* < 0.01), GBM (*P* = 0.02), KIRC (*P* = 0.02), KIRP (*P* = 0.02), LIHC (*P* < 0.001), MESO (*P* < 0.01), PAAD (*P* = 0.01), SARC (*P* = 0.02), SKCM (*P* < 0.01), UVM (*P* < 0.01) patients.

MMP13 overexpression was negatively correlated with PFS in patients with ACC (*P* < 0.001), KIRC (*P* = 0.02), KIRP (*P* = 0.04), LIHC (*P* = 0.04), MESO (*P* < 0.01), SARC (*P* < 0.01), SKCM (*P* = 0.03), UVM (*P* = 0.04) and PCPG (*P* = 0.03). Whereas increased expression of MMP13 was primarily associated with survival advantage in patients with DLBC.

Meanwhile, increased expression of the MMP13 gene in tumors predicted a poor prognosis for DSS, including ACC (*P* < 0.01), GBM (*P* < 0.01), KIRC (*P* = 0.01), KIRP (*P* = 0.01), LIHC (*P* < 0.01), MESO (*P* = 0.03), PAAD (*P* = 0.02), SARC (*P* = 0.03), SKCM (*P* = 0.02), UVM (*P* < 0.01), and HNSC (*P* = 0.04). In patients with ACC, KIRP, and SARC, high MMP13 expression predicted shorter DFI. In addition, the Kaplan-Meier analysis was applied for verification of accordingly tumors with significant prognosis (Figure 2B).

The above results showed that higher levels of MMP13 expression are associated with poorer prognosis in patients with most types of cancer.

### MMP13 DNA hypomethylation associated with poor prognosis

**Figure 3:**
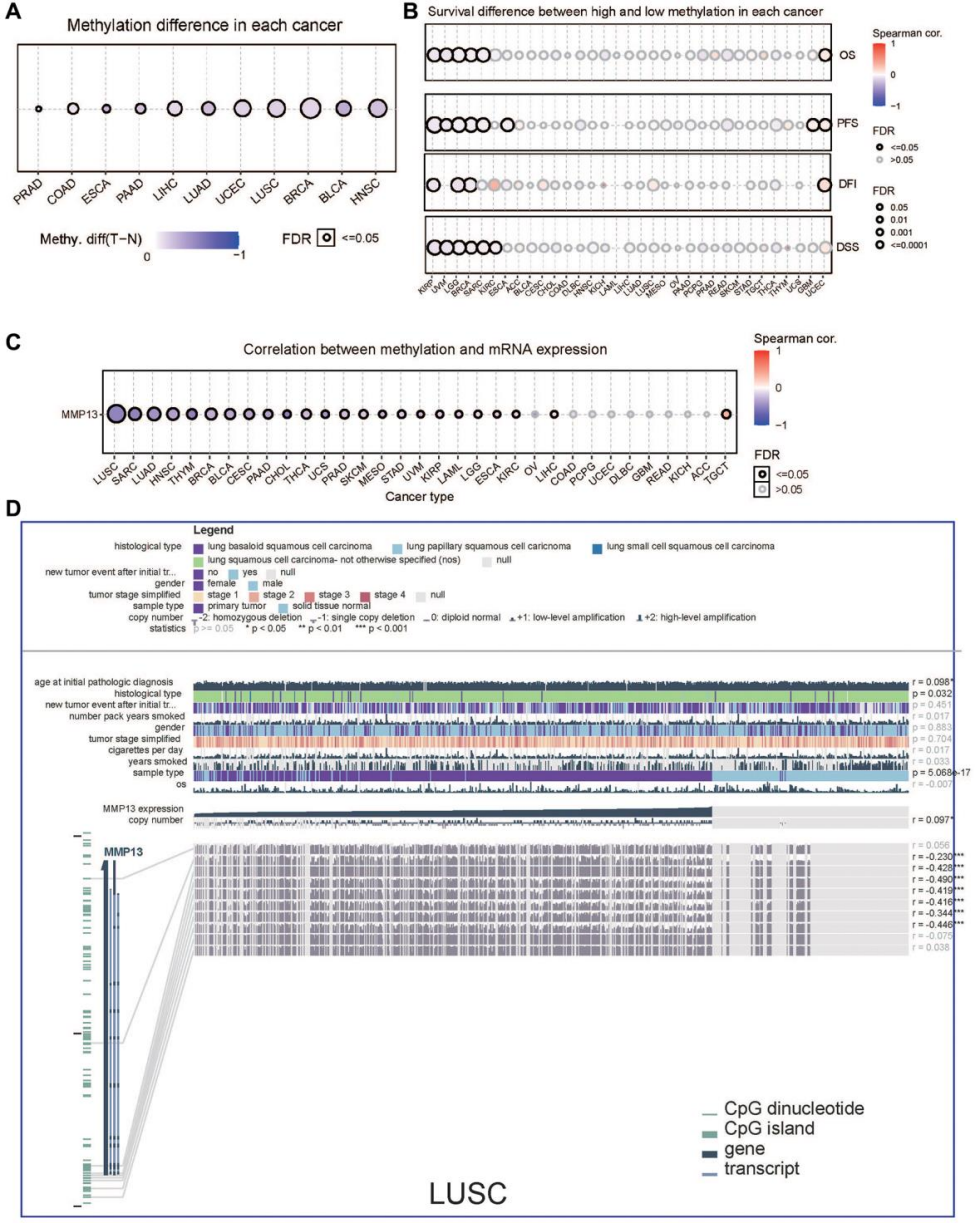
**DNA methylation analysis of MMP13 in different tumors.** (**A**) Methylation differences of MMP13 in different cancer tissues compared with normal tissues. Blue dots represent the down-regulation of methylation in tumors, and red dots represent the up-regulation of methylation in tumors; the darker the color, the greater the difference. The size of dots is positively correlated with the FDR significance. (**B**) Survival difference between MMP13 higher and lower methylation groups in each cancer. Blue dots mean they're negatively correlated, and red dots mean they're positively correlated. (**C**) Correlation between genome methylation and mRNA expression across different cancers. (**D**) This chart provides the following key information, including case-relevant clinical data, gene expression, copy number data, survival data, and DNA methylation data, along with Benjamini-Hochberg adjusted *P* value and Pearson correlation coefficient. (Statistical significance: ^*^p < 0.05, ^**^p < 0.01, ^***^p < 0.001).

DNA methylation plays a critical role in many biological activities, especially in the carcinogenic process [31]. To explore whether methylation is involved in the regulation of MMP13, we analyzed MMP13 promoter methylation levels using the GSCA database and determined the correlation between gene methylation and prognosis in 33 tumor types. MMP13 promoter methylation levels were significantly reduced in PRAD, COAD, ESCA, PAAD, LIHC, LUAD, UCEC, LUSC, BRCA, BLCA, and HNSC compared to adjacent non-tumor tissues (Figure 3A). The relationship between MMP13 promoter methylation and patient prognosis was examined (Figure 3B), in terms of OS, high MMP13 methylation levels were protective factors for patients with KIRP, UVM, LGG, BRCA, and SARC. In patients with KIRP, UVM, LGG, BRCA, SARC, and ESCA, MMP13 methylation levels were positively correlated with PFS. Regarding DFI, high MMP13 methylation levels were a protective factor in patients with KIRP, LGG, and BRCA. Analysis of DSS data showed that low levels of MMP13 methylation were associated with poor outcomes in PATIENTS with KIRP, UVM, LGG, BRCA, SARC, and KIRC.

TCGA data showed that MMP13 mRNA expression level was mainly negatively correlated with DNA methylation levels, especially in LUSC (Figure 3C). Subsequently, we performed MEXPRESS visualization analysis on 758 samples of lung squamous cell carcinoma (Figure 3D), MMP13 expression was significantly associated with the histological type (*p* = 0.032), sample type (*p* = 5.068e-17). Additionally, MMP13 expression was positively correlated with MMP13 copy number. We identified MMP13 DNA methylation at seven probes in the non-promoter region (cg03331229, cg13041032, cg19710916, cg22658979, cg19620758, cg14995062, and cg10085326) that were negatively associated with gene expression in lung squamous cell carcinoma.

### Genetic mutation analysis of MMP13 in various cancers

**Figure 4:**
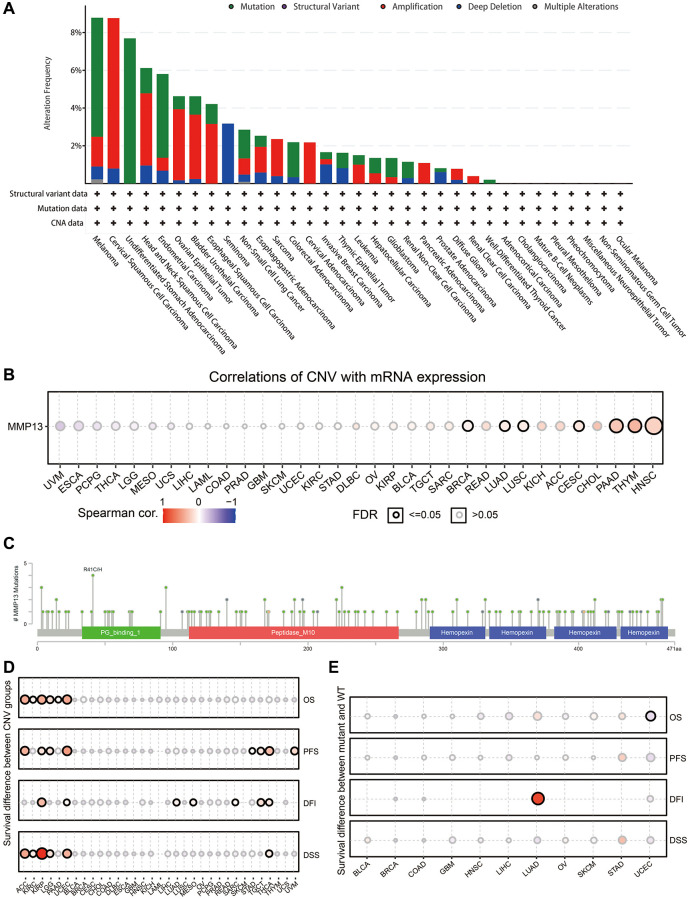
**Mutation features of MMP13 in each cancer.** (**A**) The results are displayed as a histogram of the genetic alteration type and frequency of MMP13 in each cancer. CNA, copy-number alterations. (**B**) Pan-cancer analysis of the correlation between CNV and mRNA expression of MMP13. CNV, copy number variations. CNV is equivalent to CNA in the TCGA database. Blue bubbles represent a negative correlation, and red bubbles represent a positive correlation. Bubble size correlated positively with the FDR significance. The black outline border indicates FDR ≤ 0.05. (**C**) The mutation diagram of MMP13 in pan-cancer across protein domains from the cBioPortal database. (**D**) Figure summarizes the survival difference between CNV groups in each cancer. The bubble color from blue to red represents the hazard ratio from low to high. Bubble size is positively correlated with the Cox *P* value significance. The black outline border indicates Cox *P* value ≤ 0.05. (**E**) The survival difference between mutant and wide type groups in pan-cancer. Hazard ratios and Cox *p*-values displayed by the color and size of bubbles.

We then investigated the genetic alteration frequency of MMP13 in human cancers by using the TCGA datasets in the cBioPortal database (Figure 4A). Genetic alterations in MMP13 were dominated by “amplification” and “mutation” types, which were observed in almost all TCGA cancer types. The highest frequency of MMP13 alteration (8.78%) was observed in patients with melanoma, with “mutation” as the primary type. With an alteration frequency of 8.76%, the “amplification” type predominated in the cervical squamous cell carcinoma samples. Only the “mutation” type of cancer was detected in undifferentiated stomach adenocarcinoma, showing an alteration frequency of 7.96%.

Next, the Pearson correlations between gene expression and CNV were analyzed across 33 cancer types to determine the effect of CNV on MMP13 mRNA expression (Figure 4B). It was revealed that the expression of MMP13 was positively correlated with CNV in BRCA, LUAD, LUSC, CESC, PAAD, THYM, and HNSC. Sequentially, the results revealed that missense mutation of MMP13 was identified as the primary type of genetic alteration across cancers (Figure 4C). There were 130 mutation sites detected in the location between amino acids 0 and 471, including 112 missenses, and 16 truncations, with R41C/H being the most frequent mutation site.

Additionally, the clinical survival differences in patients between CNV and wide type of MMP13 in pan-cancer samples were also investigated. CNV data from 11495 samples were downloaded from the TCGA database (Figure 4D). In ACC, KIRC, KIRP, LGG, PAAD, and UCEC, higher MMP13 copy number significantly reduced the overall survival compared to those without alteration. There were significant differences in progression-free survival between CNV and wild-type in patients with ACC, KIRP, LGG, UCEC, STAD, TCTG, THCA, and SARC. Copy number variation significantly affects the disease-free interval in patients with KIRP, UCEC, LUAD, MESO, SARC, TCTG, and THCA. Meanwhile, we also analyzed DSS and revealed that higher CNV of the MMP13 gene in tumors predicted unfavorable survival, including ACC, KIRC, KIRP, LGG, UCEC, and THCA.

Simultaneously, we analyzed the relationship between the single nucleotide variation of MMP13 and tumor prognosis across 33 cancer types (Figure 4E). The results indicated that the prognosis of most cancers was not affected by SNV, but a higher SNV of MMP13 was remarkably associated with poor DFI in LUAD and a favorable OS in UCEC patients.

These results demonstrate that CNV and mutations affect MMP13 mRNA expression in most cancer types and are closely related to patient prognosis in some specific tumors.

### Correlation analysis with TMB, MSI, tumor microenvironment, and immune markers

**Figure 5:**
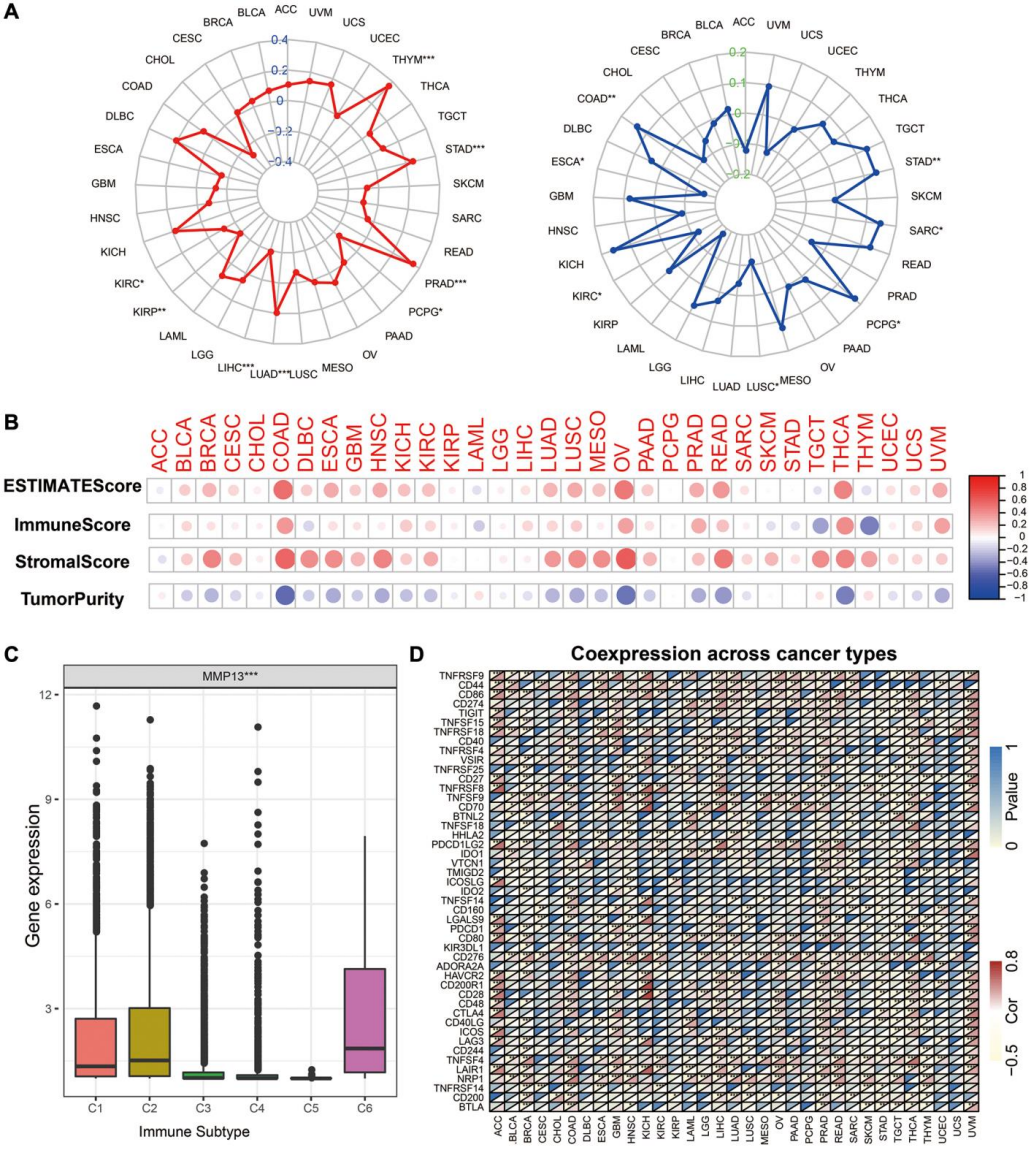
**Correlation of MMP13 expression and the TMB, MSI, and tumor immune microenvironment.** (**A**) Radar graphs display the correlation of MMP13 expression with tumor mutation burden (TMB) and microsatellite instability (MSI) in pan-cancer. The red or the blue curves represent the correlation coefficient, ^*^p < 0.05, ^**^p < 0.01, ^***^p < 0.001. (**B**) Correlation of MMP13 with the ESTIMATE score, the immune score, the stromal score, and the Tumor purity. (**C**) The differences of MMP13 expression in six immune subtypes. C1: wound healing, C2: IFN-gamma dominant, C3: inflammatory, C4: lymphocyte depleted, C5: immunologically quiet, C6: TGF-β dominant. (**D**) Heatmap illustrates the correlation between MMP13 expression and different immune genes in pan-cancer. For each pair, the top left triangle represents the *P*-value, and the bottom right triangle represents the correlation coefficient ^*^p < 0.05, ^**^p < 0.01, and ^***^p < 0.001.

Cancers with defective mismatch repair (dMMR) mechanisms often accumulate mutations in monomorphic microsatellites (short tandem repeats) and are particularly prone to mismatch errors, known as microsatellite instability (MSI). Together with tumor mutational burden (TMB), it plays a role as a predictive biomarker for immunotherapy [32]. In this study, we investigated the relationship between MMP13 and TMB, and MSI in different tumor types (Figure 5A). The results showed that the expression of MMP13 was positively correlated with TMB in THYM, STAD, PRAD, and LUAD. In contrast, MMP13 expression was negatively correlated with TMB, including PCPG, KIRP, LIHC, and KIRC. As for MSI, MMP13 expression was positively correlated with it in COAD, PCPG, SARC, and STAD and negatively correlated with it in ESCA, KIRC, and LUSC.

Based on the TCGA data, we calculated the immune, stromal, and estimate scores and tumor purity in different cancers. Then, we analyzed the correlation between MMP13 expression and the above four scores (Figure 5B). The results showed that the expression of MMP13 was significantly positively correlated with the immune score, stromal score, and estimate score in most cancer types. In contrast, there was a negative correlation with the purity tumor, which indicated that with the higher expression of MMP13, the content of immune and stromal cells was higher, reflecting that MMP13 is highly related to tumor immune microenvironment. Furthermore, we investigated the expression of MMP13 in different immune subtypes (Figure 5C). According to six different immune subtypes, including C1 (wound healing), C2 (IFN-gamma dominant), C3 (inflammatory), C4 (lymphocyte depletion), C5 (immunological quiet), and C6 (TGF-beta dominant). MMP13 expression was highest in the C6 subtype, followed by C1 and C2, and lowest in the C5 subtype. The relationship between MMP13 expression and immune-related markers in 33 tumors was further investigated. Forty-seven immune-related genes, including many immune checkpoints, such as IDO2, TMIGD2, CTLA4, etc., were significantly associated with the expression of MMP13 in most tumors.

The above results indicate that MMP13 expression is highly positively correlated with tumor immune infiltration and is a potential marker reflecting tumor immune response.

### MMP13-related protein interaction network and enriched pathway

**Figure 6:**
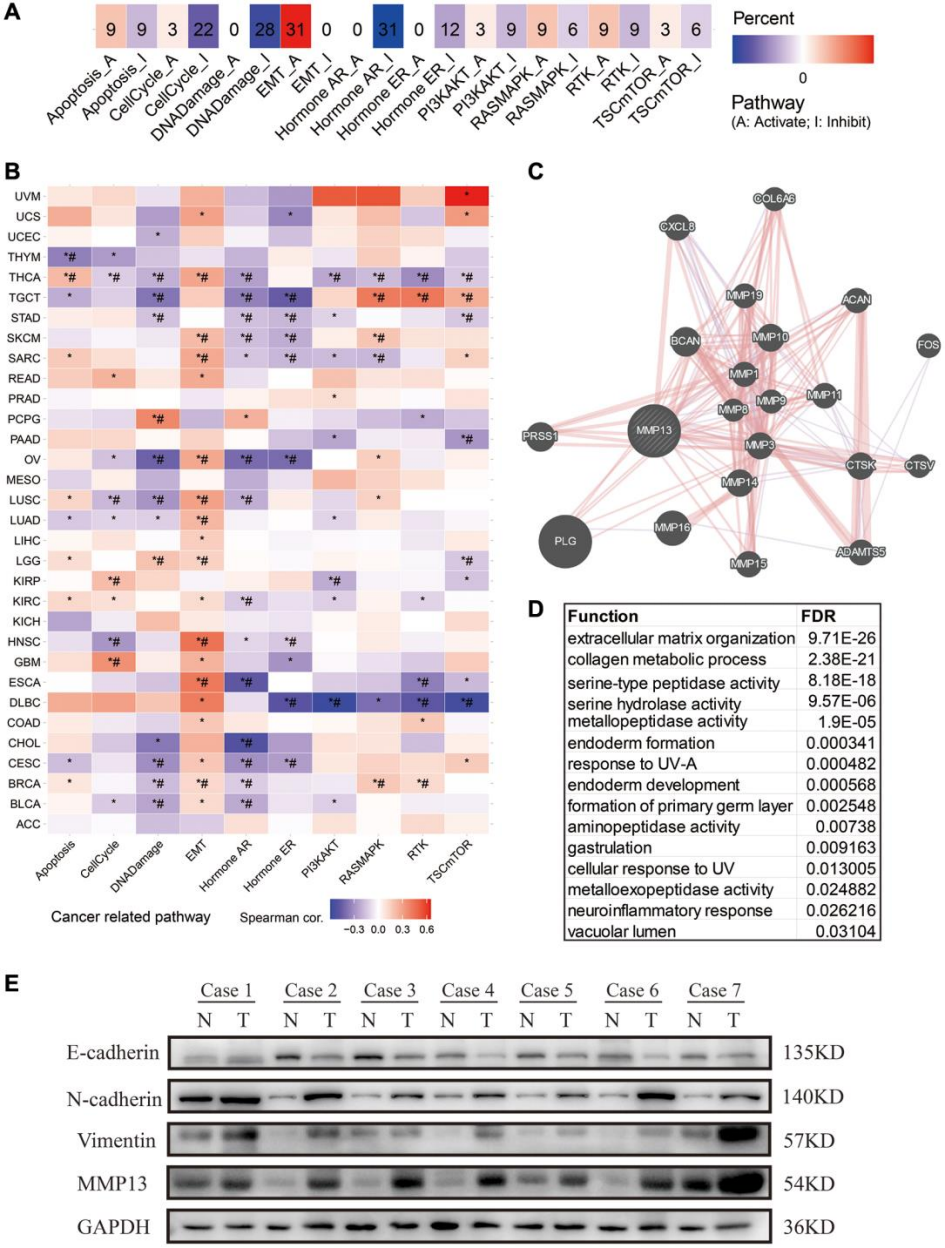
**Pathway analysis and PPI network construction for MMP13 in different tumors.** (**A**) The potential effect of upregulation of MMP13 on oncogenic pathway activity was analyzed in pan-cancer. The number in each cell represents the number of cancer types associated with the specific pathway. (**B**) The association between MMP13-based GSVA score and activity of cancer-related pathways in pan-cancers. ^*^P-value <= 0.05; ^#^FDR <= 0.05. (**C**) PPI network analysis of interacting genes with MMP13 by GeneMANIA webtool. (**D**) Functional enrichment analysis of MMP13-related genes in PPI network. (**E**) Western blotting was used to evaluate the expression of MMP13 and EMT molecular markers in 7 paired human LUAD tissues and adjacent normal tissues. N and T represented cancer tissue and adjacent normal tissue, respectively.

To explore the potential biological process of MMP13 involvement in tumorigenesis, we used the GSCA tool to investigate the correlation of MMP13 expression with the activity of cancer-related pathways (Figure 6A). The results showed that high expression of MMP13 was linked with activation of EMT pathway in approximately 31% of cancer types. The high expression of MMP13 is significantly correlated with the activation of EMT pathway in HNSC, ESCA, LUAD, LUSC, etc., as shown in Figure 6B. Furthermore, we used the GeneMANIA online program to study the potential functionally related genes of MMP13 by constructing a PPI network (Figure 6C). There are about 20 genes that are closely associated with MMP13, including PLG, MMP16, BCAN, PRSS1, MMP10, MMP14, CTSK, MMP3, CXCL8, MMP15, ADAMTS5, MMP11 ACAN, MMP1, MMP19, FOS, COL6A6, MMP9, CTSV, and MMP8. Of these, 77.64% have a physical interaction with MMP13, whereas 8.01% have a co-expression relationship. Enrichment analysis indicated that their functions are mainly related to the extracellular matrix organization, collagen metabolic process, and metallopeptidase activity (Figure 6D). For example, MMP1, MMP13, MMP14 and CTSK are involved in collagen metabolic process [33, 34]; ADAMTS5, ACAN and MMP13 are involved in the extracellular matrix organization [35–37]. MMP13, as a member of matrix metalloproteinases, may play an important role in the reorganization of extracellular matrix during tumorigenesis and the regulation of signal response in the tissue microenvironment to promote EMT. Thus, western blot (WB) was performed to examine the EMT molecules in tumor tissues. We collected LUAD tissues for WB analysis and found that MMP13 expression was increased in tumor tissues. Generally, with high expression of MMP13, the expression of epithelial cell marker E-cadherin was down-regulated, and the expression of mesenchymal cell markers N-cadherin and vimentin was up-regulated in tumor tissues (Figure 6E).

### The potential of MMP13 as a serum diagnostic marker

**Figure 7:**
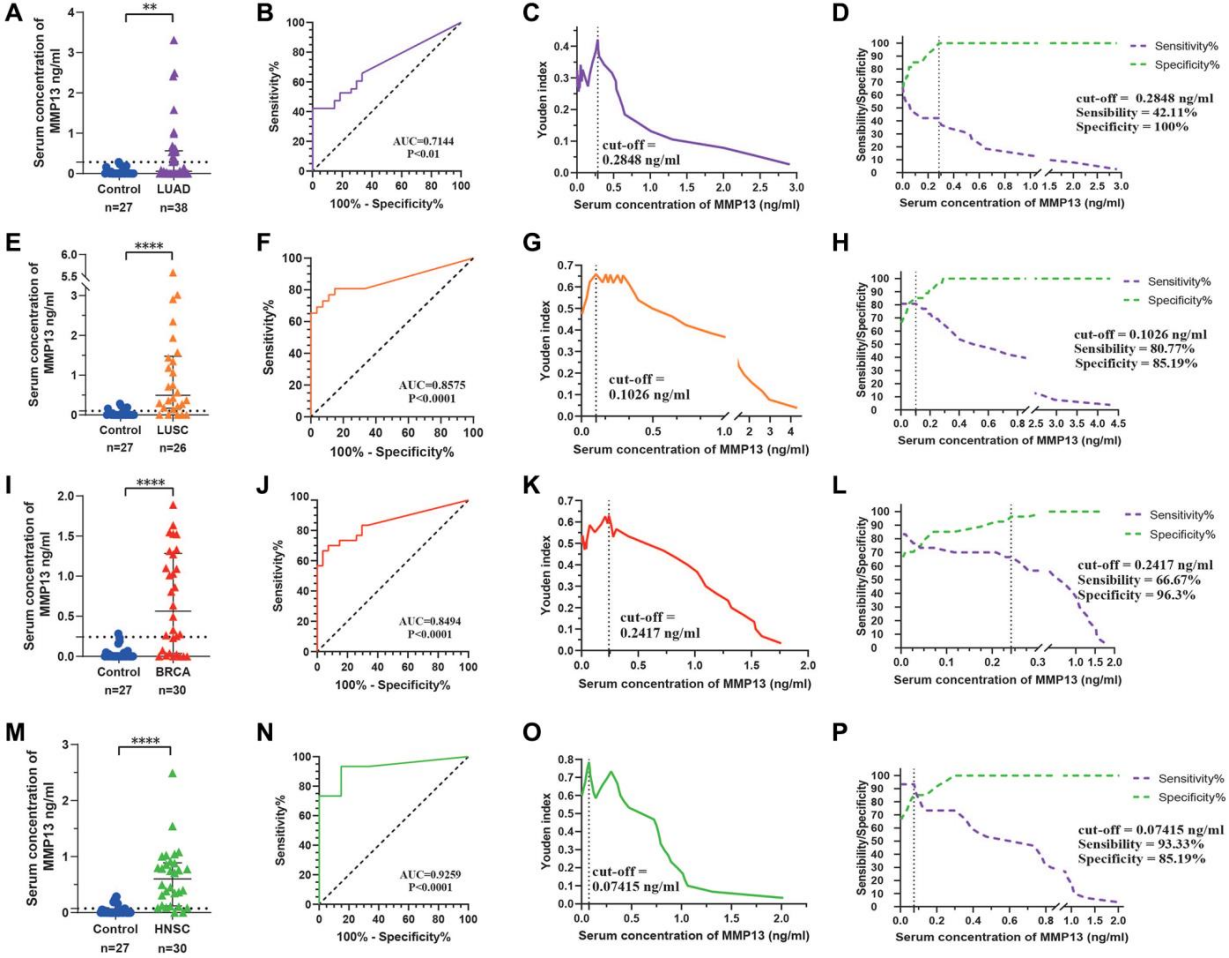
**Evaluation of the diagnostic efficacy of serum MMP13.** (**A**) Scatter plot analysis of serum concentrations of MMP13 in patients with LUAD and controls. (**B**) Serum MMP13 ROC curves for samples from patients with LUAD and control subjects. (**C**) Youden index and cut-off value in LUAD to serum MMP13 concentration. (**D**) Sensitivity and specificity in LUAD to serum MMP13 concentration. (**E**–**H**) Diagnostic tests for LUSC patients. (**I**–**L**) Diagnostic tests for BRCA patients. (**M**–**P**) Diagnostic tests for HNSC patients. The Mann-Whitney *U*-test was used for comparisons between the two groups. ^*^P < 0.05, ^**^P < 0.01, ^***^P < 0.001, ^****^P < 0.0001.

MMP13, as a secretory protein, is overexpressed in many cancers, but its expression in normal tissues is very low or nondetectable (Figure 1A, 1B), implying its potential as a serum diagnostic marker. With this in mind, we selected four tumor types with the most up-regulated MMP13, including BRCA, HNSC, LUAD, and LUSC, for ELISA verification. Compared to the healthy control group (M = 0 ng/ml, IQR: 0–0.045 ng/ml), the median concentration of MMP13 in the BRCA group was 0.56 (IQR: 0.050–1.230) ng/ml, 0.60 (IQR: 0.171–0.889) ng/ml in the HNSC group, 0.059 (IQR: 0–0.564) ng/ml in the LUAD group, and 0.495 (IQR: 0.171–1.472) ng/ml in the LUSC group (Figure 7A, 7E, 7I, 7M). ROC curve analysis showed that the serum MMP13 concentration could effectively distinguish cancer from the normal group, and the AUC values in BRCA, HNSC, LUAD, and LUSC were 0.8494, 0.9259, 0.7144, and 0.8575, respectively (Figure 7B, 7F, 7J, 7N). The concentration of MMP13 corresponding to the maximum value of the Youden index is the cut-off value for the diagnosis of this tumor. As for LUAD, the cut-off value was 0.2848 ng/ml (Figure 7C), at which the sensitivity of MMP13 for the diagnosis of LUAD was 42.11% and the specificity was 100% (Figure 7D). The cut-off values of MMP13 for diagnosing LUSC, BRCA, and HNSC and its corresponding sensitivity and specificity are shown in Figure 7G, 7H, 7K, 7L, 7O, 7P. Further, we compared MMP13 with commonly used serum biomarkers, and found that MMP13 was superior to CA15-3 (AUC = 0.6718) in the diagnosis of BRCA and CEA (AUC = 0.6915) in the diagnosis of LUAD. The diagnostic efficacy of MMP13 for HNSC was also much better than that of SCC (AUC = 0.5321), and the AUC for LUSC was slightly lower than that of conventional marker CYFRA 21-1 (AUC = 0.9444) Supplementary Figure 1. Above results demonstrate that MMP13 has an important diagnostic value in HNSC, BRCA, LUSC, and LUAD. Details of the results are shown in Supplementary Table 2.

## DISCUSSION

MMP13 belongs to the class of collagenases in the matrix metalloproteinase family and is capable of cleaving interstitial collagen as well as digesting many other ECM and non-ECM molecules [38, 39]. Previous studies have shown that MMP13 expression is upregulated in some tumors and may be predictive of poor prognosis [13, 40–43]. Pan-cancer analysis can reveal the unique and common characteristics of genes among different tumors, and discover tumor diagnostic and prognostic biomarkers. So far, no study has fully analyzed the relationship between MMP13 and tumorigenesis. In this study, we used TCGA database to perform a pan-cancer analysis of MMP13 in expression, prognosis, and biological function, revealing its value as a serum tumor marker by ELISA. MMP13 is highly expressed in multiple cancer types and tumor cell lines, whereas it was expressed at low levels in normal tissues. Survival analysis showed that MMP13 expression predicts poor patient outcomes in OS, PFI, DSS, or DFI for some types of cancer, including ACC, KIRC, KIRP, LIHC, etc. Recent studies have shown that MMP13 facilitates tumor invasion and metastasis in hepatocellular carcinoma [44]. It has also been shown that MMP13 is overexpressed in renal cell carcinoma bone metastases, predicting a poor prognosis [45]. One study suggested that MMP-13 inhibition is a useful preventive or therapeutic adjunct in colorectal cancer [46]. Consistent with this study, MMP13 may serve as an intervention target for these tumors.

DNA methylation is a chemical modification that regulates gene expression. Aberrant DNA methylation occurs in tumor cells, primarily targeting CpG islands in gene expression regulatory elements [47, 48]. Our results showed that MMP13 methylation plays a tumor-suppressing role in most cancers. And MMP13 methylation level was negatively correlated with gene expression levels, especially in LUSC (Figure 3C). Survival analysis showed that MMP13 hypermethylation was a protective factor for patients with most tumor types (Figure 3B), suggesting abnormal DNA methylation may be an important regulator of MMP13 mRNA expression in most tumor types. Genetic alterations are important factors driving the development of tumors [49, 50]. Increased frequency of MMP13 amplification increases the incidence of brain metastases in patients with lung adenocarcinoma [51]. Our results revealed that genetic alterations in MMP13 are predominantly of the “amplification” and “mutation” types in most cancer types and such genetic alterations affect the expression of MMP13 mRNA and are associated with patient prognosis in certain tumors (Figure 4). The impact of MMP13 genetic alterations on tumorigenesis, progression, and prognosis needs to be further investigated.

Immunotherapy is a breakthrough in cancer treatment [32, 52, 53]. Microsatellite instability (MSI), together with tumor mutational burden (TMB) and PD-1/PD-L1 expression, serve as predictive biomarkers for immunotherapy [54–56]. Our results showed that MMP13 was positively associated with TMB and MSI in several different tumors, suggesting that MMP13 may affect immunotherapy response. The tumor microenvironment is an integral part of cancer, and its composition has been shown to influence the response to immune checkpoint blockade (ICB) [57–61]. Based on TCGA data, we analyzed the correlation between MMP13 expression and immune, stromal and estimated scores, and tumor purity. The results showed that higher MMP13 expression was associated with higher immune and stromal cell levels and lower tumor cell levels (Figure 5B). MMP13 also strongly correlates with immune-related markers in most tumors (Figure 5D). It has been reported that MMP-9/MMP-13 in stromal and non-stromal fractions can modulate T cell responses by cleaving or shedding negative regulatory molecules associated with T cell apoptosis [62]. Therefore, MMP13 is closely related to the tumor immune microenvironment and is a potential target for tumor immunotherapy.

Epithelial-mesenchymal transition (EMT) is a key process for local or distant metastasis of cancer cells, characterized by loss of epithelial markers such as E-cadherin and upregulation of mesenchymal markers such as N-cadherin and vimentin [63]. We first found that MMP13 was associated with the activation of EMT pathway through pathway association analysis. Furthermore, we collected lung adenocarcinoma tissues as an example and confirmed that the MMP13 protein level was up-regulated in tumor tissues, while EMT-related molecules showed corresponding changes, confirming that the high expression of MMP13 was related to the activation of EMT pathway. Consistent with our findings, MMP13 has been reported to be involved in EMT in hepatocellular carcinoma, favoring invasion and metastasis [44]. We also analyzed genes that may be related to MMP13 function and found that they were mainly related to extracellular matrix organization, collagen metabolic processes, and metallopeptidase activity. (Figure 6D). As a regulator of the tissue microenvironment, the high expression of MMP13 may affect the tumor microenvironment by remodeling extracellular matrix, thus promoting the activity of EMT pathway. Therefore, the intervention of MMP13 may achieve the purpose of reprogramming the tumor microenvironment.

A particularly important finding of this study is that MMP13 can be used as a serum diagnostic marker for various tumors. We collected serum samples from patients with HNSC, BRCA, LASC, LUAD with significantly MMP13 overexpression in cancer tissues, and confirmed by ELISA that MMP13 expression was also up-regulated in the serum of patients. Based on the difference in serum concentration of MMP13, tumor and normal populations can be effectively distinguished, demonstrating its potential as a serum diagnostic marker. Moreover, MMP13 has advantages over several commonly used clinical biomarkers, such as CA15-3 for BRCA, CEA for LUAD diagnosis, and SCC for HNSC diagnosis. Previous studies have also found that MMP13 can be used as a serum marker for some tumors [7, 10, 64]. It is expected that further studies in multiple centers will be able to develop an ELISA method for above-mentioned tumor diagnosis and screening based on MMP13.

This study explored the role of MMP13 in tumor development in a comprehensive manner, showing that MMP13 is abnormally upregulated in various tumors and is closely correlated with poor prognosis, tumor immune infiltration, and EMT pathway activation. Moreover, MMP13 has the value of a serum diagnostic marker in some specific cancers. However, there are still some limitations to this study, including the lack of in-depth study on the detailed mechanism of MMP13 regulating EMT activity. The value of MMP13 as a serum diagnostic marker for some tumors has been verified by ELISA, but it is still necessary to further expand the sample size to clarify its clinical application value in the future.

## Supplementary Materials

Supplementary Figure 1

Supplementary Tables
