# Supplementary material for: Pan-cancer analysis reveals the associations between MMP13 high expression and carcinogenesis and its value as a serum diagnostic marker
Source: Aging (Albany NY). 2023 Mar 22;15(6):2115–35. doi: 10.18632/aging.204599 (PMC10085597; doi:10.18632/aging.204599)
Supplement: Supplementary Figure 1 [file aging-15-204599-s001.pdf]

SUPPLEMENTARY FIGURE

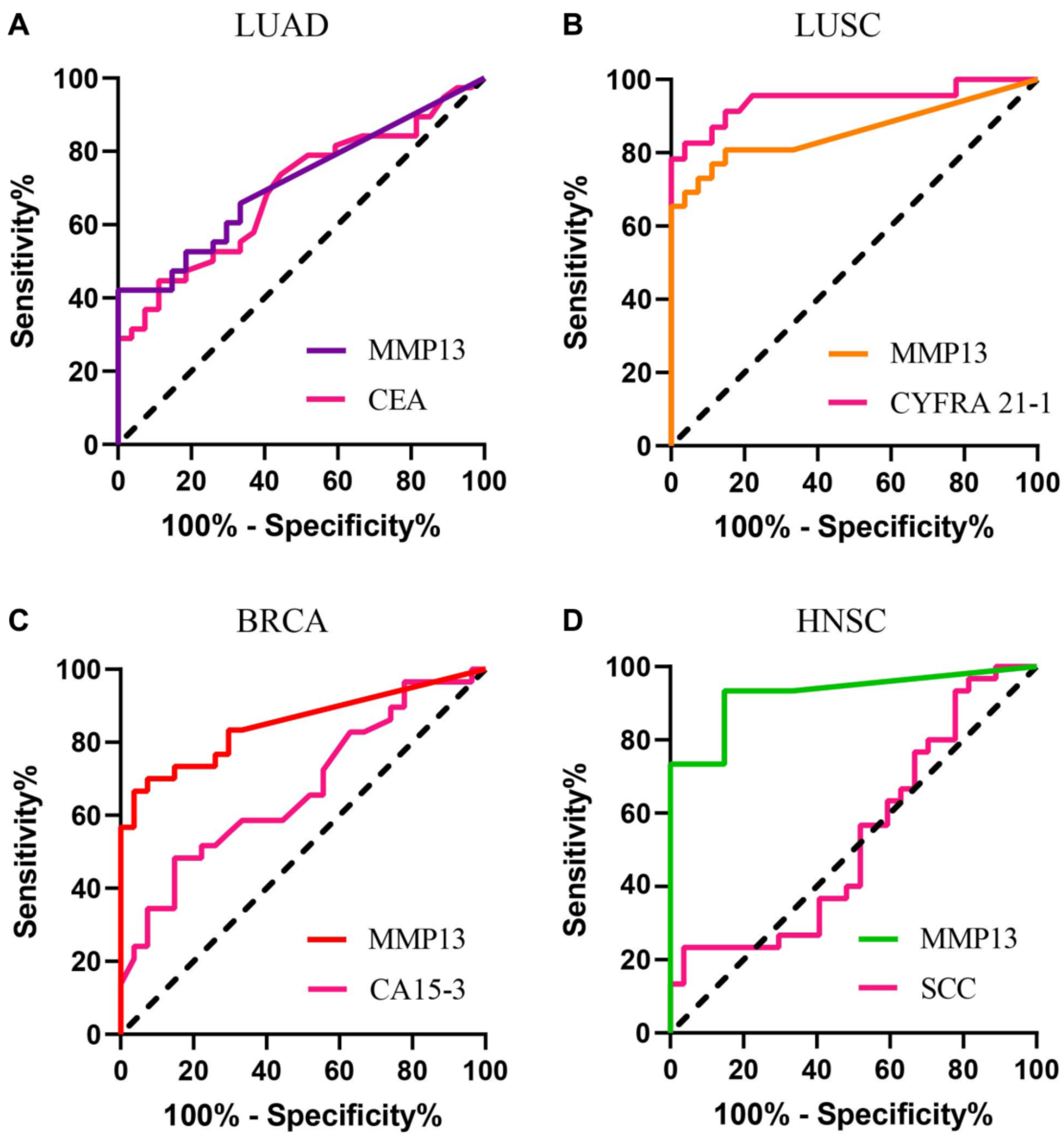

Supplementary Figure 1. The diagnostic value of MMP13 was compared with other clinical biomarkers. (**A**) Comparison of diagnostic efficacy of MMP13 and CEA in LUAD. (**B**) Comparison of diagnostic efficacy of MMP13 and CYFRA 21-1 in LUSC. (**C**) Comparison of diagnostic efficacy of MMP13 and CA15-3 in BRCA. (**D**) Comparison of diagnostic efficacy of MMP13 and SCC in HNSC.
