# Supplementary material for: Pan-cancer analysis reveals the associations between MMP13 high expression and carcinogenesis and its value as a serum diagnostic marker
Source: Aging (Albany NY). 2023 Mar 22;15(6):2115–35. doi: 10.18632/aging.204599 (PMC10085597; doi:10.18632/aging.204599)
Supplement: Supplementary Tables [file aging-15-204599-s002.pdf]

SUPPLEMENTARY TABLES

Supplementary Table 1. Antibodies used in Western blot analysis.

Name of antibodies	Catalog number
E Cadherin (6B10) Mouse mAb	ZEN-BIOSCIENCE 201283
N Cadherin Rabbit pAb	ZEN-BIOSCIENCE 380671
Vimentin (D21H3) XP® Rabbit mAb	Cell Signaling Technology #5741
Anti-GAPDH Mouse Monoclonal Antibody	Transgen HC301-01
MMP13 Rabbit Polyclonal antibody	Proteintech 18165-1-AP
Goat Anti-Rabbit IgG H&L (HRP)	ZEN-BIOSCIENCE 511203
Goat Anti-Mouse IgG (H+L) HRP	SparkJade EF0001

Supplementary Table 2. Diagnostic efficacy of MMP13 in ELISA.

	Number	Median (ng/ml)	AUC	Cut-off (ng/ml)	Sensitivity (%)	Specificity (%)	Diagnostic efficacy of clinical biomarkers
Healthy control	27	0 (IQR:0–0.045)					
BRCA	30	0.56 (IQR: 0.050–1.230)	0.8494	0.2417	66.67	96.3	CA15-3: 0.6718
HNSC	30	0.60 (IQR: 0.171–0.889)	0.9259	0.07415	93.33	85.19	SCC: 0.5321
LUAD	38	0.059 (IQR: 0–0.564)	0.7144	0.2848	42.11	100	CEA: 0.6915
LUSC	26	0.495 (IQR: 0.171–1.472)	0.8575	0.1026	80.77	85.19	CYFRA 21-1: 0.9444
